# Supplementary material for: Are carbon emissions trading and green financial instruments synergistic? -Comprehensive quantitative research based on content analysis
Source: PLoS One. 2024 Mar 7;19(3):e0298601. doi: 10.1371/journal.pone.0298601 (PMC10919601; doi:10.1371/journal.pone.0298601)
Supplement: S1 Data — (ZIP) [file pone.0298601.s001.zip › Data/地级市Z_F工作报告.zip!/║╙─╧/╓ú╓▌╩╨/└┤╫╘╣1⁄2╓┌║┼í╛┬φ┐╦╩2╛▌═°í┐_2017.docx]

各位代表：

现在，我代表市人民政府向大会报告工作，请予审议，并请各位政协委员和其他列席人士提出意见。

一、2016年工作回顾

2016年是我市现代化进程中具有重要意义的一年。市十一次党代会提出了“率先在全省全面建成小康社会，加快现代化进程，向国家中心城市迈进”的奋斗目标，为我市未来发展绘就了蓝图、指明了方向。过去的一年，面对国内外复杂多变的发展环境和经济下行压力持续加大的严峻形势，在省委、省政府和市委的坚强领导下，全市上下牢记使命、同心同德，深入贯彻习近平总书记系列重要讲话精神，认真落实中央、省委、市委重大决策部署，牢固树立新发展理念，主动适应经济发展新常态，统筹推进“四大重点”工作，经济社会持续平稳健康发展，较好完成了市十四届人大三次会议确定的目标任务，实现了“十三五”良好开局。

一大批事关全局的节点性、标志性、奠基性大事要事取得重大突破，加快现代化进程、在全省率先全面建成小康社会的基础更加坚实、支撑更加有力、前景更加广阔！

——发展质量效益稳步提高。初步统计，全市生产总值完成7994.2亿元、增长8.4%，分别高出全国、全省1.7个、0.3个百分点，省会经济首位度19.9%；地方财政一般公共预算收入突破千亿元大关、达到1011.2亿元、增长14.3%，高出全省6.3个百分点，占全省的32.1%；固定资产投资完成6998.6亿元、增长11.3%；社会消费品零售总额完成3665.8亿元、增长11.3%；进出口总值完成550.3亿美元，占全省的77.3%，继续位居中部省会城市首位，为全省发展大局作出了积极贡献!

——战略支撑更加坚实。中国（郑州）跨境电子商务综合试验区、郑洛新国家自主创新示范区、中国（河南）自贸区、国家大数据综合试验区、国家通用航空产业综合示范区、国家综合交通枢纽示范城市等国家战略规划和平台相继落地，郑州的国家政策叠加优势在全国城市中屈指可数。尤其是国家明确提出支持郑州建设国家中心城市，必将对郑州乃至河南的发展产生重大而深远的影响!

——产业转型升级步伐加快。以先进制造业为支撑、以现代服务业为主导的现代产业体系加快构建，三次产业比由2015年的2.1∶49.5∶48.4调整为2.0∶47.3∶50.7，第三产业占比超过第二产业，实现了历史性突破。

——综合交通枢纽地位持续强化。郑徐高铁通车运营，郑万、郑合高铁加快建设，郑济、郑太高铁开工建设，米字形高铁网加快成形。新郑机场旅客吞吐量首次突破2000万人次、居国内机场第15位，货运量45.7万吨、跃居国内机场第7位。

——畅通郑州工程建设取得重大进展。轨道交通2号线一期投入运营、城郊铁路一期和1号线二期开通试运营，运营总里程达到93.6公里。西三环北延、东三环高架、农业路快速化、南三环高架东延二期等重大交通工程建设取得突破，中心城区“井字+环线”快速路网体系基本形成。

——群众生活水平不断改善。精准扶贫扎实有效，全市脱贫3.39万人、退出贫困村119个，荥阳、新郑、中牟全部脱贫，脱贫攻坚三年行动计划大头落地；公共财政继续向民生领域倾斜，民生支出1031.2亿元、增长18.4%，占公共财政预算支出的78%，省市民生实事全面完成；城镇居民人均可支配收入33214元、增长6.8%，农民人均可支配收入18426元、增长7.6%。人民群众获得感持续增强。

一年来，我们重点做了以下工作：

（一）调结构、转方式，现代产业体系加快构建。把调结构作为产业转型升级的主攻方向，聚焦提质增效，着力推动结构调整和产业转型升级。新型工业化加快推进。扎实推进电子信息、汽车、高端装备等产业基地建设，规模以上工业增加值完成3215.4亿元、增长6%，七大工业主导产业完成工业增加值2294.9亿元、增长7.8%，汽车产业首次突破千亿元，千亿级产业集群达到5个。高新技术工业增加值占工业增加值的43%，成为制造业发展的重要力量。“互联网+”指数居全国城市第14位，获批开通国际通信专用通道，入围中国信息化城市15强。现代服务业增长迅猛。现代金融、商贸物流、文化创意旅游等提质增速，服务业增加值完成4057.1亿元。郑东新区金融集聚核心功能区累计入驻金融机构288家，中原金控、九鼎金融租赁等新兴非银行金融机构迅猛发展，成功举办首届中国（郑州）国际期货论坛，金融集聚核心功能区的影响力和辐射力进一步增强。全市金融业增加值占现代服务业的20%。电子商务完成交易额4900亿元、增长33%。会展经济稳步发展，入选中国会展最具办展幸福感城市。以创建“国家全域旅游示范区”为带动，接待国内外游客8933.6万人次、增长13.2%，旅游总收入1053.9亿元、增长13.7%。市场外迁成效显著，市场资源有效整合，新建成市场集聚区1046.5万平方米，社会累计投资达500亿元。现代都市农业加快发展。国家级、省级龙头企业分别达到13家、62家，农业产业化集群达到29个。首批6万亩都市生态农业示范园基本建成。新增可追溯标准化“菜篮子”生产基地2万亩，新建和改造提升标准化农贸市场30家，农产品质量安全监测合格率稳居全国前列。产业集聚区和服务业“两区”加快建设。全市产业集聚区对工业增长、投资拉动的贡献率分别达到51.1%、35.6%；服务业“两区”主营业务收入完成1544.9亿元、增长10%，从业人员达到31万人。郑东新区中央商务区成为全省首个六星级服务业“两区”。

（二）抓关键、激活力，重点领域改革持续深化。聚焦落实标志性、关键性改革举措，发展内生动能进一步释放。围绕供给侧结构性改革，突出“去降补”政策落实，减少无效和低端供给，原煤、钢材产量分别下降21.7%和14.5%；关闭煤矿61家，占全省的61%；市区商品住房去库存任务全部完成，价格过快上涨势头得到初步遏制，房地产市场宏观调控成效显著；企业降成本有力有效，累计为企业减负45.3亿元。围绕破解体制机制瓶颈，国家“互联网+政务服务”示范工程成功申建，进一步简政放权，对160项行政审批事项中涉及的中介服务事项进行全面梳理。“五证合一、一照一码”“先照后证”全面实行，信息共享全面推进，事中事后监管不断加强，商事登记制度改革深入推进。围绕优化市场要素配置，营改增试点全面推开，涉企资金基金化改革和金融体制改革深入推进。新增新三板挂牌公司60家、占全省41%，全市挂牌上市公司达到184家，新增资本市场融资600亿元。扎实推进投融资体制改革，进一步拓展社会资本投资渠道和领域，加快推动政府和社会资本合作模式项目落地。制定实施市级禁止类和限制类清单，开展企业投资项目管理负面清单试点，PPP模式投资1818亿元，已落地699亿元。地方政府融资平台市场化转型取得积极进展。围绕解决民生需求，农村综合改革、文化、教育和医药卫生体制改革等事项稳步推进。国企改革、财税体制改革持续深化。

（三）提品质、扩成效，新型城镇化建设协调推进。把“以建为主、提升品质、扩大成效”作为战略重点，加快推进城市现代化国际化、县域城镇化和城乡一体化。新型城镇化新三年行动计划全面实施。郑东新区、上街区、高新区、中原区、金水区基本实现全域城镇化，全市城镇化率71%。登封、新密入选国家新型城镇化第三批综合试点。大棚户区改造和安置房建设成效突出，货币化安置率逐步提高，74个安置房项目启动桩基施工，49个安置区实现群众回迁，累计回迁群众13.2万人。82个“三级三类”市民服务中心开工建设。入选国家传统村落名录4个、省级传统村落名录16个。城市交通基础设施建设扎实推进。以轨道交通为重点的公交都市建设加快推进，轨道交通3号线一期、5号线等进展顺利，快速公交形成“5主65支”运营网络；嵩山南路、花园路等一大批道路完成整修。智慧交通建设取得突破。新建公共停车泊位7.1万个。中心城区承载能力持续增强。新建改建供水管网125公里，新增燃气用户12万户、供热面积1052万平方米，侯寨水厂、双桥污水处理厂建设顺利推进，马头岗污水处理厂二期和郑州新区污水处理厂基本建成投用。入选国家综合管廊试点城市和省级海绵城市试点城市。城市精细化管理持续提升。以整治“四乱”为抓手，以“四区一环十五路”为重点，强力推进631条支路背街综合整治，城市环境较好改善。

（四）植优势、增后劲，开放水平和创新能力持续提升。大力推动开放创新，以开放创新促转型、促发展。开放水平不断提升。航空港实验区加快建设，机场二期全面建成投用，开通客运航线186条、全货机定期航线34条；以智能终端等产业集群为抓手，手机总产量2.6亿部，全球重要的智能终端（手机）研发制造基地初具规模；成功获批全国双创示范基地；通航“郑州制造”、郑州航展渐成品牌，通航产业发展基地逐步形成。郑州新郑综保区三期封关运行，进出口总值居全国海关综保区第一位。中欧（郑州）班列开行251班，载货量、满载率等综合实力持续位居全国前列。汽车、肉类、活牛、邮政等特种功能性口岸稳定运营，粮食、药品等口岸加快建设，河南国际贸易“单一窗口”上线运行，跨境电商交易额实现翻番。经开综保区成功获批。大力开展精准招商，引进市外资金1680亿元，实际利用外资37.2亿美元。科技创新取得重大突破。荣获国家促进科技和金融结合试点城市，高新区被认定为国家首批科技服务业试点区域，获批国家知识产权示范园区，企业孵化能力跃升至全国第2位。政府主导的20个创新创业综合体建成527.6万平方米，全市孵化器、众创空间达到151家。中科院过程所郑州分所、郑州信大先进技术研究院、三磨所国家重点实验室等建设稳步推进。“智汇郑州·1125聚才计划”年度任务圆满完成。科技进步对经济增长贡献率达61%。

（五）重防治、促统筹，生态文明建设扎实推进。坚持发展、保护两手抓，综合施策、标本兼治，强力推进生态建设和环境治理。蓝天工程深入实施。坚持大气污染防治深化、细化、常态化，统筹推进燃煤、扬尘、机动车等污染治理，全市空气质量优良天数达到159天、同比增加21天，PM10、PM2.5平均浓度分别下降14.4%、18.8%，综合指数在全国74个重点监测城市排名退出后五位，全省大气污染防治工作年度考核获得优秀、名列前茅。生态绿化扎实推进。植树造林10.3万亩，市区新建绿地1529万平方米，“两环三十一放射”建成生态廊道362公里、累计绿化面积3261万平方米；建成公园游园34个，第十一届园博会园博园、南水北调生态文化公园等加快建设，“一环一渠三网”生态景观带基本建成。生态水系加快建设。贾鲁河综合治理、牛口峪引黄工程开工建设，环城生态水系循环、石佛沉砂池至郑州西区生态供水工程加快推进。农村人居环境持续改善。创建省级生态县（市）1个、生态乡（镇）2个、生态村6个。

（六）惠民生、增福祉，社会事业全面进步。坚持发展为民，弥补民生短板，努力实现经济与社会发展相互促进、良性互动。就业和社会保障工作持续加强。全市新增城镇就业14.9万人，农村劳动力转移就业9.5万人；发放创业担保贷款10.6亿元。城镇居民医疗保险与“新农合”实现并轨，城乡居民养老保险参保228.8万人；首批机关事业单位养老保险参保登记21.6万人。城乡低保、农村五保连年提标，全年保障8.8万人，实现动态管理下的应保尽保。新开工棚改住房12.4万套，保障性住房基本建成5.2万套；落实公租房预分配制度，分配入住1万多套。社会事业全面发展。全市新建、改扩建幼儿园39所；市区新建、改扩建中小学校36所，新增学位5万余个；市区义务教育阶段学校接收随迁子女新生入学5万余人。一大批优质医疗资源项目投入使用。新增社会办养老机构8家、新建社区养老服务中心示范点20个，新增养老床位2600多张。“四个中心”建设加快推进，现代公共文化服务体系建设迈出新步伐，公共文化设施实现全覆盖。谋划启动商都历史文化区、古荥大运河文化区等文化片区建设；配合文化部推动“二十四节气”成功申报联合国教科文组织人类非物质文化遗产代表作名录。成功举办中欧政党高层论坛经贸对话、丙申年黄帝故里拜祖大典、国际少林武术节、国际旅游城市市长论坛、首届中国考古学大会和首届“楚河汉界”世界棋王赛等大型活动。我市荣膺“全国双拥模范城”七连冠。积极防控风险维护稳定。更加注重用法治思维和法治方式推进社会建设。平安郑州建设扎实推进，视频监控体系基本实现“全覆盖、无盲区、大通联、高效用”。打击严重暴力犯罪绩效、破获命案积案全国领先。信访维稳形势总体向好，突发事件应急处置能力持续提升。重拳打击和分类处置非法集资，扎实开展安全生产大检查、打非治违和专项整治，全力保障食品药品安全，社会大局和谐稳定，人民群众安全感和满意度持续提升。

（七）转作风、提效能，政府自身建设不断加强。深入开展“两学一做”学习教育，严格执行中央八项规定精神及省市若干意见，全市会议费下降48.7%，公车购置及运行费下降60.8%。自觉接受人大监督、政协民主监督和社会监督，办理省人大建议、省政协提案62件，市人大议案6件、建议565件，市政协提案801件，除《关于加强城市管理提高城市管理水平的议案》市人大常委会未通过外，其他议案、建议和提案全部办结，满意率99%以上。加强重点领域立法，切实提升政府立法水平，制定政府规章5部，提请市人大常委会审议地方性法规2部。严格落实“一岗双责”，持续整治懒政怠政为官不为，强化行政监察和审计监督，加大廉政建设和反腐败工作力度，专项治理发生在群众身边的不正之风和腐败问题，一批违法违纪人员受到惩处。

加强社会主义核心价值观教育，社会文明程度不断提高。国防教育、国防后备力量及人民防空建设持续加强，我市被评为“全国人防工作先进城市”。外事、侨务、对台、民族、宗教、统计、档案、史志、气象、地震、社科、红十字会、对口支援新疆哈密等工作，都取得了新的成绩。

各位代表！过去一年成绩来之不易，这是党中央、国务院，省委、省政府和市委坚强领导的结果，是市人大、市政协大力支持的结果，是全市人民奋力拼搏的结果。在此，我代表市人民政府，向广大工人、农民、知识分子、企业家，向人民解放军指战员、武警官兵、公安民警及社会各界人士致以崇高敬意！向关心支持郑州发展的港澳台同胞、海外侨胞和国际友人表示诚挚感谢！

我们也清醒认识到：发展不足仍是郑州最大的实际，保持经济较快发展面临较大压力；产业核心竞争力不强，新旧动力转换仍需时日，转型升级任务艰巨；在更宽领域、更高层次参与全球资源要素整合的能力还不强，国际竞争力、国际知名度还有待进一步提升；创新基础相对薄弱，高层次的科教机构和创新型、开放型人才不足；城乡发展方式粗放、城市综合承载力不足，基础设施建设仍滞后于城市的快速发展，城乡二元结构、“大都市病”问题亟待解决；生态环境约束加剧，大气、水、土壤污染等环境问题仍然突出，系统推进绿色低碳发展的措施还不够；与人民群众生活密切相关的就业、教育、医疗等公共服务供给还没有得到有效解决；政府职能转变仍需深化，依法行政水平、政务服务效率、社会治理能力和城市管理能力亟待提升；一些领域的不正之风和腐败问题尚待根除。对此，我们一定千方百计加以解决，决不辜负人民的期望和重托！

二、2017年工作指导思想和主要预期目标

今年是党的十九大召开之年、供给侧结构性改革的深化之年，是我们贯彻落实国家对郑州新定位、新规划的起步之年，做好政府工作意义重大。

综合判断，今年外部环境依然错综复杂，世界经济仍在深度调整，不稳定不确定因素增多，总体仍延续疲弱复苏态势。国内经济运行也存在不少突出矛盾和问题，产能过剩和需求结构升级矛盾突出，经济增长内生动力不足，金融风险隐患有所聚集等，都将对我们的发展带来一定影响。但同时我们也要看到，中央应对能力强、政策回旋余地大，保持宏观政策连续性，深入推进供给侧结构性改革，出台促进中部地区崛起“十三五”规划、中原城市群发展规划，明确提出支持郑州建设国家中心城市，为我们提供了有利的发展氛围和难得的政策机遇；经过近一个时期尤其是“十二五”以来的发展，我市交通区位、人力资源、产业基础、开放载体平台等传统优势加速向综合优势转化。新一轮科技革命和产业变革蓬勃兴起、供需关系正在发生深刻变化，为我们推进产业发展和结构调整提供了更多的市场机会。市十一次党代会提出了“一枢纽一门户一基地四中心”的奋斗目标，确立了“四重点一稳定一保证”的工作总格局，全市上下形成了心齐、劲足、风清、气正的良好氛围。只要我们牢牢把握这一轮郑州发展的重要战略机遇期，持续强化龙头、引领和支撑“三大作用”，真抓实干、负重前行，就一定能够推动经济社会持续健康较快发展，在全省、全国大局中抢占发展制高点、赢得战略主动权！

今年政府工作的指导思想是：全面贯彻党的十八大和十八届三中、四中、五中、六中全会精神，更加紧密地团结在以习近平同志为核心的党中央周围，深入贯彻习近平总书记系列重要讲话精神和党中央治国理政新理念新思想新战略，落实习近平总书记调研指导河南提出的打好“四张牌”要求，统筹推进“五位一体”总体布局和协调推进“四个全面”战略布局，以新发展理念为引领，以提高发展质量和效益为中心，以推进供给侧结构性改革为主线，以建设国家中心城市为统揽，持续深化“四重点一稳定一保证”工作总格局，持续坚持目标导向和问题导向及“创优势、增实力、补短板、能抓住”工作方针，把握“稳中求进”总基调、“奋发有为”总要求，突出项目带动、项目化推进，统筹做好稳增长、促改革、调结构、防风险、惠民生各项工作，努力实现经济平稳健康发展和社会和谐稳定，以优异成绩迎接党的十九大胜利召开。

主要预期目标是：生产总值增长9%左右，一般公共预算收入增长9%左右，固定资产投资增长12%左右，规模以上工业增加值增长8%左右，社会消费品零售总额增长10%左右，进出口总值增长3%左右，实际利用外资基本持平，常住人口城镇化率提高1.6个百分点左右，城乡居民收入增长与经济增长基本同步，居民消费价格涨幅2%左右，节能减排、环保等约束性指标完成上级下达任务，发展质量效益明显提高，结构性改革取得实质性进展。

贯彻指导思想，实现预期目标，做好政府工作，我们将坚定不移地做到“五个必须”。一是必须切实增强“四个意识”，坚决维护党中央权威。始终在思想上、政治上、行动上同以习近平同志为核心的党中央保持高度一致，切实增强政治意识、大局意识、核心意识、看齐意识，严守政治纪律，坚守政治定力，坚定跟着党的令旗走，党中央制定的方针政策必须执行，党中央确定的改革方案必须落实。二是必须坚持新发展理念，以发展理念转变引领发展方式转变。适应引领把握发展新常态，把“创新、协调、绿色、开放、共享”新发展理念落实到经济社会发展全过程、各领域、各环节，努力走出一条更高质量、更有效率、更加公平、更可持续的发展道路。三是必须坚持“以人民为中心”的发展思想，增进人民福祉、促进人的全面发展。政府一切工作都是为了人民，受人民之托、为人民打工。把增进人民福祉、促进人的全面发展作为发展的出发点和落脚点，抓住群众最关心最直接最现实的利益问题，带着责任和感情做好民生工作，持续提高人民群众的幸福指数，让人民在共建共享中有更多获得感。四是必须坚持改革发展稳定协调推进，营造良好发展环境。持续深化供给侧结构性改革，坚决打好“三去一降一补”攻坚战；全面深化重点领域和关键环节改革，充分释放发展动力和活力；持续强化社会治理创新，坚持底线思维，着力防范化解各类风险，努力维护社会大局和谐稳定。五是必须坚持以项目为抓手，统筹推进“四大重点”工作。突出项目带动、项目化推进，通过项目推进促建设、强投资、调结构、惠民生、求提升、转作风，通过项目推进实现“四大重点”工作的新突破，实现郑州综合承载力、产业竞争力、开放带动力、创新驱动力、文化影响力和社会治理能力全面提升。

三、2017年重点工作

做好今年的政府工作，我们将按照市十一次党代会确定的“四重点一稳定一保证”的工作格局，统筹谋划、协同推进、综合施策、精准发力，努力完成全年目标任务。重点抓好以下工作：

（一）科学推进新型城镇化，着力为国家中心城市建设筑牢空间支撑。紧紧抓住国家中心城市建设重大机遇，瞄准目标，正视差距，坚定信心，加强对指标体系、发展路径、战略重点等发展规划的研究，高起点规划、高标准建设、高效率推进。当前，我们将围绕“城市现代化国际化、县域城镇化、城乡一体化”主线，按照“以建为主、提升品质、扩大成效”的要求，不断提升城乡环境质量、群众生活质量和城市综合竞争力。

加快推进城市现代化、国际化。坚持规划引领优化布局，强化城市设计，推动城市设计精品化、人文化，加快推进主城区有机更新、生态修复、内涵提升，加快东部新城区、南部新城区、西部新城区开发建设，打造复合型现代化新城区。进一步强化国际化现代化立体综合交通枢纽地位。继续加快国家航空一类口岸、铁路一类口岸和国际陆港、多式联运体系建设，配合做好米字形高铁、机场至高铁南站等城际铁路建设，构建枢纽型、功能性、网络化的现代综合交通运输体系。持续推进畅通郑州工程。以城市道路交通关键节点工程建设为带动，力争开工建设6号线、8号线一期等8条轨道交通线路；加快推进5号线、3号线等6条线路建设；开工建设四环快速化等一批重大交通工程，确保东三环（107辅道）高架部分、南三环东延二期等建成通车；加快中原西路等22条常规公交专用道建设，新增新能源或清洁能源公交车500台以上，公共交通机动化出行分担率达到国内城市一流水平。大力实施棚户区改造。严格按照安置房建设的目标任务，坚持高标准建设，新开工棚户区改造住房10.3万套以上，基本建成保障性住房7万套，进一步提高货币化安置率，确保今年回迁群众35万人以上。让更多回迁群众告别棚户区，让广大人民群众在住有所居中创造新生活！全面提升城市品质。围绕宽带中国、智慧城市、“互联网+”及大数据等试点载体，加快推进“城市大脑”建设。以新型智慧城市、海绵城市、地下管廊建设为引领，统筹地下建设与地上建设，解决“逢雨必涝”“马路拉链”等群众反映强烈的现实矛盾和问题；加大城市给排水、电力、燃气、供热等建设力度，拓展和提升城市综合承载力。加快建设商都历史文化区、古荥大运河文化区、二砂文化创意园、百年德化文化复兴工程等一批历史文化街区、文化景观和国际化风情小镇，抓好市民公共文化服务区等重点片区建设。深化城市管理体制改革，坚持标准化、精细化、品质化、智慧化贯穿城市管理全过程，提升城市设施和人居环境的现代化、国际化水平。大力开展爱国卫生运动和健康城市建设，确保顺利通过国家卫生城市届满复审和全国文明城市考评。深化区域联动发展与合作。突出郑州在中原城市群中的核心带动作用，推进与开封、新乡、许昌、焦作等周边毗邻城市融合发展，加快基础设施互联互通，推动资源共享、生态共建、产业互动，着力构建组合型大郑州都市区，为加快形成带动周边、辐射全国、连通国际的中原城市群核心区奠定坚实基础。

加快推进县域城镇化。深入贯彻全省百城建设提质工程动员会精神，着力抓好培育主导产业、提高综合承载力、改善公共服务水平等关键环节，按照卫星城的标准，加快新郑、新密、荥阳、登封等试点城市提质工程建设。更加注重以人为本，完善土地、财政、社保等配套政策，实现农民和市民权益互联互通，让农村转移人口“进得来、留得住、过得好”。统筹推进县城交通道路、水电气暖、垃圾和污水处理等基础设施建设，逐步强化县城、组团新区以及产业集聚区的城市功能。加快推进区位明显、基础较好、潜力较大的18个中心镇建设发展，增强集聚功能，发挥节点支撑作用。大力发展县域经济，积极承接产业转移，培育壮大产业集群，促进形成资源集约、产城互动、生态宜居、文明和谐的新型城镇。

加快城乡一体化发展步伐。因地制宜、量力而行，先期培育一批产业形态特色鲜明、美丽环境和谐宜居、传统文化彰显特色、设施服务便捷完善、体制机制充满活力的特色小镇。健全城乡一体化体制机制，促进城乡公共资源均衡配置，推进基本公共服务和社会服务网络向农村覆盖，形成结构合理、区域协调的城乡一体化发展新格局。持续改善农村人居环境，以实施农村生活垃圾分类集中处理工程为抓手，通过市县两级共同治理，建立起“村收集、乡转运、县（市）集中处理”的垃圾处理系统；大力实施农村生活污水集中处理工程，加大投入，分期建设，努力实现生活污水集中处理全覆盖。持续开展美丽乡村建设试点，着力建设一批“宜居、宜业、宜游”美丽乡村，努力实现村村有特色，处处有美景！

（二）扎实推进结构调整，着力为国家中心城市建设夯实产业基础。聚焦主导产业和实体经济，顺应需求特别是消费需求新趋势，以加快新旧动能转换、提升供给体系质量效率为中心，加快结构调整步伐，向结构要市场、向结构要动力、向结构要效率。

做强先进制造业。适应市场变化，立足产业基础，加大投资力度，坚定不移调高技术水平、调强制造能力、调优产业结构、调长产业链条，以提高质量和核心竞争力为中心，建设中国制造强市。大力推进电子信息、汽车、装备制造、生物医药等七大产业基地建设，力争电子信息产业产值达到3500亿元，汽车产业产值达到1200亿元，装备制造业产值达到2000亿元。改造提升铝及铝精深加工、现代食品、品牌服装及现代家居等传统产业，重塑传统产业发展新优势。积极创建“中国制造2025”城市试点，全面提升郑州制造竞争力，启动市级制造业创新中心建设，重点在高端装备、新材料等领域争取1～2家企业列入省级制造业创新中心培育企业。加快“中国地铁产业城”建设，积极筹建信息安全产业园，加快中原物联网体验中心建设，努力争取国家智能制造专项和试点示范项目。

做大现代服务业。加快推进金融机构集聚和服务模式、产品、业态创新，继续办好中国（郑州）国际期货论坛，支持郑州商品交易所拓展新的交易品种，促进发展大宗商品国际贸易，提升以郑东新区为主体的区域性金融中心集聚辐射功能。依托国家现代物流创新发展试点城市建设，不断完善国际航空物流园区、国际陆港等示范园区功能，培育电子商务、快递等行业物流。推进国家服务外包示范城市建设，大力培育服务贸易和服务外包本土品牌。加快推进大围合区域市场外迁工作，进一步整合市场资源，增强核心业态的竞争力和影响力。继续做好“国家全域旅游示范区”创建工作，支持登封建设中国少林功夫国际旅游目的地，推进文化旅游产业加快发展。紧紧把握“房子是用来住的、不是用来炒的”定位，坚持因地施策，库存量大的区域把去库存和促进人口城镇化结合起来，去化周期短的区域坚持“稳价放量”，加大土地供应，强化市场监管，促进房地产市场平稳健康发展。以“文化创意+”为引领，大力发展休闲娱乐、影视动漫等创意产业。扩大服务业消费需求，重点支持科技服务、金融服务、通用航空、服务外包、健康养老、家居服务等新兴产业，推广文化、体育、绿色等消费模式。力争新增挂牌上市公司30家以上，电子商务交易额和网络零售额增长18%，服务业增加值增长10.5%，固定资产投资增长16%。

做优现代都市农业。以农业供给侧结构性改革为主线，培育农业农村发展新动能，提高农业综合效益和竞争力。围绕创建国家农业可持续发展试验示范区，进一步提升农业的生态保障功能，围绕主城区周边调整农业种植结构，大力发展花卉、苗木、林果、牧草等生态农业和生态林业，着力构建主城区生态圈。把增加绿色优质农产品供给放在突出位置，进一步增强“菜篮子”保障能力，深入推进都市生态农业示范园建设，着力打造农业产业化集群，力争新培育市级产业化龙头企业10家、市级以上集群达到30个，实现休闲农业营业收入35亿元。积极配合办好全国农产品加工业发展和农业品牌创建推进大会。

大力发展网络经济。贯彻落实网络强国战略、国家大数据战略及国家“互联网+”行动计划，以统筹电子政务集约化建设为抓手，深化信息技术在经济社会各领域的广泛应用，推进网络强市建设。大力发展大数据经济、智慧经济、分享经济、创意经济等新经济，加快推进郑东新区龙子湖大数据产业基地、高新区大数据应用产业园建设，着力打造国家大数据综合试验区核心区。加快“北斗云谷”千亿科技城建设。重塑产业链、供应链、价值链，拓展发展新经济，加速制造业智能化服务化、现代都市农业智慧化精准化、服务业品质化高端化，让产业拥抱互联网，插上腾飞的翅膀！

全面加强质量和品牌管理。以提高质量和核心竞争力为中心，强力推进标准引领、质量提升、品牌带动“三大工程”，建设质量强市。弘扬企业家精神，引导企业发扬工匠精神，启动郑州工业品牌认定工作，推动郑州速度向郑州质量转变、郑州制造向郑州创造转变、郑州产品向郑州品牌转变。

加快推进产业集聚区和服务业“两区”建设。实施产业集群提质、重大项目增效、科技创新提升、要素支撑保障、产城互动发展“五大工程”，稳步提升产业集聚区建设水平，力争全年固定资产投资达到2800亿元，实现规模以上工业主营业务收入8500亿元。推动服务业“两区”壮大产业规模，形成集群发展优势，提升业态档次，增强品牌集聚和市场竞争力，力争固定资产投资完成400亿元，超百亿元产业集群达到6个。

（三）持续推进开放创新，着力为国家中心城市建设集聚发展动能。把开放作为在经济下行情况下抢占制高点、提升竞争力的重要抓手，把创新作为发展动力转换、调整经济结构的根本路径，以开放促创新，以创新促开放，着力拓展发展空间，增强发展动能。

作为不临海、不临江、不沿边的内陆城市，我们一定要以海纳百川的姿态，高效务实的作为，敢为人先的锐气，勇于担当的干劲，围绕建设中西部对外开放门户，深度融入“一带一路”倡议，充分发挥国家战略政策叠加优势，大力发展更高层次的开放型经济！

强力推进航空港实验区建设。以“龙头带动、集群配套、创新协同、链式发展”为路径，以八大产业集群为抓手，大力发展以智能终端产业为代表的现代产业基地，力争智能手机产量达到3亿部，企业主营业务收入突破4000亿元，超百亿元工业企业4家以上。大力推进全国双创示范基地建设，尽快形成一批可复制、可推广的“双创”经验，着力打造“中国创客城”。

大力推进自贸区建设。积极推进商事、监管、金融“三项制度创新”和投融资、贸易、物流、监管服务、人员往来“五大便利化”。加快推进自贸区制度改革、体制机制创新和综合服务大厅、国际贸易“单一窗口”建设，深入推进关检合作，积极推行负面清单，扎实做好上海自贸区“28+6+19”成功经验复制推广，着力把郑州片区建设成为中国（河南）自贸区全方位深化改革引领区、多领域扩大开放先行区和中部地区经济社会与政府转型的示范区。

大力发展枢纽经济。加快国际航空货运枢纽建设，组建基地航空公司，增开国际航线，增加国际航班班次。密切与国际陆港、中欧（郑州）班列协调合作，实现“一带一路”国家和地区物流体系全覆盖，力争机场旅客吞吐量2250万人次、货运量53万吨。大力发展通用航空，以郑州通航试验区为基地，以郑州航展为品牌，构建以上街机场为平台的通航机场群网，加快国家通用航空产业综合示范区建设。尽快启动国际陆港二期建设。加快中欧（郑州）班列覆盖北欧线和南欧线的核心枢纽建设，实现班列线路持续延伸和拓展，逐步将班列物流优势转化为国际贸易优势，力争全年开行300班以上。着力促进海关特殊监管区整合提升，推动郑州新郑综保区转型升级，力争年度出单量突破180万单；加快整合出口加工区和河南保税物流中心，推进经开综保区验收运营。大力推进跨境电子商务综合试验区建设，加快线上“单一窗口”综合信息服务、线下综合园区、人才企业孵化“三大平台”建设，着力打造互联网+商贸流通现代化示范区。

大力发展口岸经济。加强口岸经济研究，强力推动口岸经济发展。加快汽车、肉类、澳洲活牛、粮食等口岸配套设施建设，推动水果、冰鲜水产品和食用水生动物等口岸年内实现规模化运营，争取药品、植物种苗等口岸申建获批。完善“一站式”大通关服务体系，打造“四港联动、多式联运”综合服务平台；加强与丝绸之路经济带沿线城市、长江经济带、京津冀和东北地区等跨区域关检合作，推动海关“一带一路”区域通关一体化和检验检疫全国通关一体化。

持续开展精准招商。围绕主导产业定位，瞄准国内外500强和行业龙头企业，借助黄帝故里拜祖大典、豫港经贸合作等重大活动，谋划招商引资项目不少于100个。全面落实“五职”招商责任制，确保73个“五职”项目当年签约、当年开工。积极招大引强选优，力争全年新引进“四力”型项目50个、引进市外域内资金增长5%。加快优化开放环境，在法定范围内制定招商引资优惠政策，提高综合服务能力，加快打造市场化国际化法治化营商环境，让郑州成为权力下放最多、管的最少、服务最优的投资热土！

同时，大力实施 “走出去”“引进来”战略。鼓励优势企业到境外开展产能、装备制造合作、承包工程等，实施跨境并购，参与国际竞标。围绕城市国际化，有序推进服务业对外开放，推进科技、教育、文化、医疗卫生等领域开放合作。

把创新放在发展全局的核心位置，以郑洛新国家自主创新示范区建设为统领，深入推进大众创业万众创新，加快打造中西部地区创新高地。

加快国家自主创新示范区建设。全面加快推进实施体制机制改革和政策先行先试，以“一区四园”为载体和龙头，谋划建设“中原科创谷”，力争示范区内万人发明专利拥有量达到27件，技术合同成交额年增长12%以上，高新技术产业产值占规模以上工业总产值60%以上。

突出科技创新的支撑作用。围绕产业链部署创新链，在我市智能终端、轨道交通装备、精密机械制造、超硬材料、北斗导航、大数据、物联网、新能源汽车、新材料、信息安全等领域，实施一批重大科技专项；依托盾构及掘进技术国家重点实验室、中船重工713所、中国电子科技集团第27研究所、郑州机械研究所、郑州大学等科研院所和高等院校组建产业技术创新战略联盟，培育一批科技创新龙头企业，突破一批关键共性技术，促进产业转型升级，提高产业发展支撑力，推动产业向价值链中高端迈进。强化创新主体培育，支持企业组建国家和省级研发机构，引导企业加大研发投入，增强技术研发能力，形成技术领先优势，培育科技型企业达到3000家，新增高新技术企业150家，实现高新技术产业产值突破8000亿元。推动国家专利审查协作河南中心、国家技术转移河南中心、国家知识产权服务业集聚发展试验区、中科院软件所郑州分所等项目建设。积极做好大连理工大学重大装备制造郑州研究院、华中科技大学郑州智能装备研究院等项目签约并启动建设。认真谋划中科院电工所、中科院光电研究院等中科院系统院所和中国空间技术研究院来郑设立分支机构，力争新建产业技术创新研究院3个。发挥创新创业综合体示范带动作用，在政府主导的20个综合体中建立科技综合服务平台，实现与部门、孵化器（众创空间）、高校、科研院所、企业之间的交流互动。加快科技成果转移转化，推动区域协同创新发展。

加强科技人才队伍建设。深入实施“智汇郑州·1125聚才计划”，力争全年新引进领军人才和高层次紧缺人才200名、领军型创业团队20个，汇聚 “两院”院士、国家“千人计划”、“万人计划”等顶尖人才20名，认定一批行业技术领域院士工作站。加强与科技部对接，筹划建立国家科技领军人才创新驱动中心。大力实施本地优秀人才素质提升、全民技能振兴、海外人才聚焦三大工程，加快形成具有郑州特色的人才政策体系。

（四）突出生态建设和环境治理，着力为国家中心城市建设提供宜居宜业环境。以“大生态、大环保、大格局、大统筹”为基本要求，坚持“治”“建”并举，坚持源头治理和末端治理并重，深入实施生态环境治理三年行动计划，着力构建森林、湿地、流域、农田、城市五大生态系统，加快建成国家生态文明先行示范区。

持续开展大气污染防治攻坚战。牢固树立打“持久战、攻坚战”意识，努力推动防治工作从末端治理向源头减排的转变。在继续强化末端治理的同时，围绕源头治理实施“四个一批”，即淘汰一批、转型一批、转移一批、替代一批。大力推进气代煤、电代煤，全市燃煤锅炉基本完成拆改，削减煤炭消费总量，煤炭占能源消费总量比重降到65%以下。坚决淘汰落后产能，推进钢铁、水泥、化工等重点行业清洁生产。实施第五阶段国家机动车污染物排放标准；完成黄标车和老旧车辆淘汰任务，市政环卫车辆优先更新为新能源或清洁能源汽车，市区60%的燃油公交车更换为新能源或清洁能源汽车；全面遏制扬尘污染，强化施工监管，必须做到开工前“六个到位”、施工中“七个100%”，按规定安装远程视频监控和PM10现场检测设备并接入市监控中心；强化餐饮油烟、垃圾治理；持续做好秸秆禁烧和烟花爆竹禁放工作。完善重污染天气应急应对机制，依据预警等级，迅速启动应急预案，落实应急措施。推进全民治污、依法治污、科学治污、精准治污，确保完成省定目标任务。治理雾霾人人有责，贵在行动、成在坚持。我们相信，通过全社会不懈努力，蓝天必定会一年比一年多起来！

切实抓好水生态文明建设。响应省政府提出的“郑州城区几条河流三年内能下河游泳”的指示，坚决打好水污染防治攻坚战，坚持“全域水系、循环水系”理念，以水量倍增和扩水增湿工程为重点，积极推进生态水源工程，完成环城生态水系循环工程、石佛沉砂池至郑州西区生态供水工程建设；牛口峪引黄工程主体基本建成，西水东引等水源工程加快推进。强力推进贾鲁河、索须河综合治理等河湖生态景观提升工程，实施郑东新区水系连通和新郑市水系连通工程；启动南水北调中线新郑段、航空港区段调蓄工程；加快黄河湿地公园后续工程、郑汴中央湿地公园等项目建设，启动荥阳、中牟、郑东新区、金水区、惠济区等沿黄有关区域的湿地修复建设。强力攻坚，抓好河流截污治污工程，把确保水质达标、治理黑臭水体、保障饮水安全作为攻坚重点，建立“河长制”，一河一策、精准治污、水陆统筹，系统推进水污染防治、水生态保护和水资源管理工作。

持续加快生态绿化建设。围绕重塑“绿城”形态风貌，扎实推进中心城区绿色生活圈、城区外围生态隔离圈、市域边界森林组团防护圈、黄河沿岸森林屏障带“三圈一带”和“一环、一路”生态景观带建设，完成森林资源培育和林业产业种植11.5万亩。加快侯寨森林公园、郑州市森林公园、石桥湿地公园、中牟黄河湿地鸟类保护区等大型主题森林公园和湿地公园（保护区）建设。启动建设14个生态保遗项目。建成园博园，精心筹办第十一届园博会；扎实推进中心城区“300米见绿、500米见园”公园绿地建设工程，建成公园游园24个，市区新建绿地800万平方米以上。我们将努力积累更多生态财富，构筑可持续发展的绿色长城！

同时，大力推进土壤污染治理，实施化肥、农药使用量零增长行动，开展土地重金属污染治理和修复，保护提升耕地质量。

全面节约和高效利用资源。研究制定能源总量预算管理实施办法和预留指标管理办法，探索建立碳排放权初始分配制度和交易市场，加强能源总量和能耗强度双控，落实国家、省统一部署的主要污染物排放总量控制，配合做好碳排放权初始分配，鼓励企业积极参与全国、全省碳排放权交易，推动用能权有偿使用和交易试点相关工作开展。持续推进工业、交通、建筑、公共机构等重点领域节能减排。积极发展循环经济和高效、低耗、绿色、环保的生态产业，推动环境保护与经济发展相生共赢。

（五）全面深化重点领域和关键环节改革，着力为国家中心城市建设激发强大活力。围绕破解经济社会发展中的深层次问题、处理好政府和市场关系，充分发挥市场在资源配置中的决定性作用和更好发挥政府作用，实施一批标志性、引领性、突破性的重大改革举措，使改革红利加快转化为发展活力。

深化供给侧结构性改革。继续实施《郑州市推进供给侧结构性改革总体方案（2016—2018年）》，全面落实农业、制造业、服务业供给侧结构性改革专项行动方案，进一步梳理推进“三去一降一补”需集中出台和落实的改革举措，打好组合拳，使各项改革措施同向发力，努力实现有效供给水平明显提高，过剩产能稳妥有效化解，落后产能基本淘汰，实体经济活力明显增强，各类风险得到有效管控，基础设施和民生事业短板得到有效弥补，结构调整取得重大进展，经济质量和效益显著提高，发展的平衡性、包容性、可持续性不断增强。

持续深化政务体制改革。加大“放、管、服”力度，启动实施政务服务集成化提供改革。加快市级政务服务集中办事大厅建设，力争10月份投入运行。推进实体办事大厅与网上服务平台融合，加快政务信息资源互认共享，力争年底前全市50%以上的政务服务事项实现网上办理。健全完善并联审批机制，将与建设项目并联相关的水、电、气、暖等公共服务事项一并纳入并联审批。深化行政审批制度改革和清单制度改革，进一步“减证便民”，优化审批流程和环节，提高审批效能。加快行政审批服务标准化建设，加强中介服务管理，试点推行“同城通办”，全面推行政务服务“一口受理”、四级联动，坚决除烦苛之弊、施公平之策、开便利之门，着力打通“最后一公里”，让群众共享改革成果！

深化投融资体制改革。明确政府投资范围，建立定期评估调整机制，优化投资方向和结构，依法发起设立政府引导、市场化运作的各类投资基金，鼓励政府和社会资本合作，发挥政府投资的引导和带动作用。确立企业投资主体地位，试点企业投资项目承诺制，探索以政策性条件为引导、企业信用承诺、监管有效约束为核心的管理模式。依托多层次资本市场，大力发展直接融资，支持有真实经济活动支撑的资产证券化，优化金融资源配置，更好服务投资兴业。

全面深化国有企业改革。以增强企业活力为中心，以提高国有资本配置和运营效率为目标，分类推进市属国有企业改革。商业一类国企实行公司制股份制改革，13家市属工业国有企业全面实现国有股权整体退出，僵尸企业及时出清；商业二类国企，保持国有资本控股地位，支持非国有资本参股；平台类公司向投资控股公司或资本经营公司转变；公益类国有企业采取国有独资经营或推进投资主体多元化方式进行改革。加快探索市管企业中市管人员和市场化聘用人员的分类管理，推行市场化、契约化、扁平化管理，不断增强国有经济和国有企业的综合实力。

加快推进农村产权改革。研究落实承包土地“三权分置”办法，完成农村土地承包经营权确权登记颁证工作。稳步推进农村集体经营性资产权股份合作制改革，健全农村产权交易服务体系，积极开展农村产权交易工作。继续深化水利、林业、供销等领域改革。

同时，我们将加快推进信用体系建设，优化企业投资经营环境，深入推进财税体制、商事制度、金融支持小微企业等改革。我们将毫不动摇地鼓励支持引导非公有制经济发展，引导民间资本优先进入先进制造业、基础设施、现代服务业、现代农业、信息产业等领域。进一步完善知识产权保护等方面的政策措施，增强非公有制经济发展信心。我们将着力营造“亲”“清”政商关系，构建政府履职尽责、支持民营企业发展、民企遵纪守法、自觉规范经营的良好局面！

（六）切实保障和改善民生，着力为国家中心城市建设营造和谐稳定社会环境。以“人民对美好生活的向往”为目标，以实施重点民生工程为抓手，加快健全基本公共服务，持续增强人民群众现实获得感。

强力推进脱贫攻坚。完善扶贫攻坚联动机制，坚持问题导向，聚焦薄弱环节，推动精准脱贫各项政策措施落地生根。落实“两年脱贫攻坚、后续巩固提升”的要求，完成易地扶贫搬迁4800人、整村推进20个，在全省率先实现“贫困村全部退出、扶贫对象全部脱贫”，确保扶贫对象与全市人民一道迈入全面小康！

稳定扩大就业。就业是最大的民生。实施就业优先战略，激发大众创业活力，推动创业带动就业。加强对灵活就业、新就业形态的支持，突出做好高校毕业生、去产能企业分流职工、农村富余劳动力三大群体就业工作，做好军转干部、退役士兵接收安置工作，做好农民工返乡创业、城镇就业困难人员和登记失业人员等帮扶工作。发放创业担保贷款10亿元，新增城镇就业13万人、农村劳动力转移就业7万人，零就业家庭动态为零，城镇登记失业率控制在4%以内。我们将牢牢抓住就业这一民生之本，让人民在劳动中创造财富，在奋斗中实现人生价值！

织密社会保障网。全面完成机关事业单位养老保险制度改革，整合城乡居民医疗保险，适度提高基本养老金标准、城乡居民医保财政补助和个人缴费标准。围绕法定人群全覆盖，实施全民参保登记行动计划，扩大社会保险参保覆盖面。进一步提高城乡低保、农村五保等补助标准，落实特困人员供养制度，切实兜住困难群体民生底线。认真做好农村“三留守”人员关心关爱工作。支持社会福利和慈善事业发展，大力发展老龄事业。持续深化计划生育服务管理改革，确保全面两孩政策平稳实施。

繁荣发展社会事业。全力推进学前教育惠民发展工程，继续实施学前教育三年行动计划。加快普及高中阶段教育。推动义务教育均衡发展，通过市区两级联动，有序做好市区高中外迁和中小学建设工作，确保新建、改扩建市区中小学校30所、新增学位3万个以上。发展现代职业教育，依托已成立的两个职教集团，深化校企合作。推动高等教育加快发展，支持郑州大学创建“双一流”大学。通过与国内外知名高校合作办学，加快培养我市急需的航空、高端制造、金融、物流等专业人才。我们要大力发展人民满意的教育，以教育现代化支撑城市现代化，使更多孩子成就梦想、更多家庭实现希望！加快区域医疗中心建设，着力打造国家区域儿童医疗中心。继续深化医药卫生体制改革和计划生育服务管理改革，在推进公立医院改革、推动分级诊疗、发展社会办医、促进优质资源下沉、保障妇幼健康等方面迈出更大步伐。大力推进国家食品安全示范市创建。紧紧围绕华夏历史文明传承创新中心建设目标，继续深化国家公共文化服务体系示范区建设，深入推进文化惠民工程，大力实施文艺精品创作工程，完善市场体系，壮大文化产业，加快市民公共文化服务区“四个中心”、郑州数字文化馆、公共文化数字云平台和城市24小时自助图书馆等项目建设，努力使文化发展成果覆盖城乡、惠及全民。大力发展新型全民健身设施和体育场地建设，打造城市社区“15分钟健身圈”，使更多人享受运动快乐、拥有健康体魄！

防范化解各类风险隐患。加快平安郑州、法治郑州建设，更加注重用科技、法治和创新手段抓好社会治理，建立立体化、信息化社会治安防控体系。时刻绷紧安全生产弦，突出道路交通、消防火灾、食品药品等重点领域综合治理，加强防灾减灾救灾工作，确保人民群众生命财产安全。深化信访制度改革，压实信访工作责任，引导群众依法理性有序表达诉求。加强互联网管理，持续打造清朗网络空间。建立完善社会风险评估机制，加强矛盾纠纷排查化解，努力从源头上预防群体性事件和个人极端案（事）件的发生，确保社会大局和谐稳定。

我们将以群众最急、最愿、最盼为先导，努力办好交通出行、市容环境、就业创业、教育医疗、食品安全、大气污染防治等十件重点民生实事，让城市更美好，市民更舒心，百姓更幸福！

加强国防动员、国防后备力量和人民防空建设，推动军民融合深度发展。支持驻郑解放军、武警部队建设，深化“双拥”共建工作。依法管理民族宗教事务，促进民族团结进步。全面做好统计、审计、地方志、外事侨务、对台事务、档案管理、文物保护、防震减灾、对口支援新疆哈密等工作。支持工会、共青团、妇联、工商联、科协、文联、社科联等群团组织工作。

四、加强政府自身建设

加快建设国家中心城市，迫切需要我们主动顺应时代发展新趋势，牢固树立新发展理念，准确把握郑州发展新方位，不断满足人民群众新期待，提升郑州治理体系和治理能力的现代化水平，加快建设法治、高效、廉洁政府。

（一）坚持依法行政，建设法治政府。全面贯彻国家法治政府建设实施纲要和郑州实施方案，坚决落实中央关于各级政府重大决策出台前向同级人大报告的规定，把依法行政、依法办事贯穿政府决策、执行全过程。加大政务公开力度。创新普法教育机制，深入推行“七五”普法，强化政府工作人员尊法学法守法用法意识，坚持科学决策、民主决策、依法决策，自觉运用法治思维和法治方式深化改革、推动发展、化解矛盾、维护稳定，营造更加公平、正义、开放的发展环境，让法治的阳光照耀政府的权力，照亮每个公民的生活！

（二）全面履职尽责，建设高效政府。推进“两学一做”学习教育常态化制度化，把履职尽责、落实见效贯穿工作始终，全面提升政府效能和工作水平。坚持守土有责，守土尽责，面对矛盾敢于迎难而上，面对失误敢于承担责任，坚持实干苦干，对于确定的工作任务和目标，说干就干，干就干好。严格监督考核，加强激励约束，强化失职追责，推动各级公务人员全面履职，担当尽责。同时，建立容错纠错免责机制，为敢于担当的干部担当，为敢于负责的干部负责。认真落实公务员分类改革政策，持续深化公务员教育培训，推动政府工作人员加强学习、提高素质，切实提高履职尽责能力，与人民群众同心协力，以实干推动发展，以实干赢得未来！

（三）持续改进作风，建设廉洁政府。把纪律和规矩挺在前面，认真执行《关于新形势下党内政治生活的若干准则》和《中国共产党党内监督条例》。严格执行中央八项规定精神和省市若干意见，驰而不息纠正“四风”，确保“三公”经费零增长。巩固专项治理成果，深化懒政怠政、为官不为等集中专项治理，抓好整改规范、重点评议、建章立制等工作，确保各项治理工作取得实效。保持清正廉洁本色，落实党风廉政建设主体责任和“一岗双责”，持续正风肃纪，加强行政监察和审计监督，重拳整治侵害群众利益的不正之风和腐败问题，以廉洁从政的实际成效取信于民。

各位代表！作为人民的政府，我们一定自觉接受人民的监督，依法接受人大及其常委会法律监督和工作监督，主动接受政协民主监督，严格执行人大及其常委会决定，认真办理人大代表建议议案和政协委员提案，广泛听取民主党派、工商联、无党派人士、人民团体和社会各界意见，自觉接受社会监督和舆论监督，让行政权力始终在法治轨道下运行！

各位代表！梦想照亮未来，奋斗铸就辉煌。郑州发展已站在新的历史起点上，让我们更加紧密地团结在以习近平同志为核心的党中央周围，在省委、省政府和市委的坚强领导下，不忘初心、继续前进，务实重干、克难攻坚，以豪情开启新征程，以实干谱写新篇章，为持续增进民生福祉，为率先在全省全面建成小康社会，加快现代化进程，加快推进国家中心城市建设而努力奋斗，以优异成绩向党的十九大胜利召开献礼！
